# miR-27a rs895819 Polymorphism and Recurrent Pregnancy Loss in Caucasian Women: A Novel Genetic Risk Factor in a Challenging Fertility Dilemma

**DOI:** 10.3390/cimb47040271

**Published:** 2025-04-11

**Authors:** Georgia Panagou, Anastasios Potiris, Dimitra Dedousi, Despoina Mavrogianni, Ioanna Vassilaki, Athanasios Zikopoulos, Efthalia Moustakli, Antonios Sfakianakis, Nikolaos Kathopoulis, Angeliki Gerede, Periklis Panagopoulos, Ekaterini Domali, Peter Drakakis, Sofoklis Stavros

**Affiliations:** 1 First Department of Obstetrics and Gynecology, Alexandra Hospital, Medical School, National and Kapodistrian University of Athens, 115 28 Athens, Greece; zetat_@hotmail.com (G.P.); dimitradedousi@hotmail.com (D.D.); dmavrogianni@med.uoa.gr (D.M.); jeannettevas@windowslive.com (I.V.); nickatho@gmail.com (N.K.); kdomali@yahoo.fr (E.D.); 2 Third Department of Obstetrics and Gynecology, University General Hospital “ATTIKON”, Medical School, National and Kapodistrian University of Athens, 124 62 Athens, Greece; thanzik92@gmail.com (A.Z.); perpanag@med.uoa.gr (P.P.); pdrakakis@med.uoa.gr (P.D.); sfstavrou@med.uoa.gr (S.S.); 3 Laboratory of Medical Genetics, Faculty of Medicine, School of Health Sciences, University of Ioannina, 451 10 Ioannina, Greece; thaleia.moustakli@gmail.com; 4 Homerton Fertility Centre, Homerton University Hospital, London E9 6SR, UK; antsfak@gmail.com; 5 Department of Obstetrics and Gynecology, Democritus University of Thrace, 691 00 Campus, Greece; agerede@otenet.gr

**Keywords:** miR-27a gene, rs895819 polymorphism, recurrent pregnancy loss (RPL), miscarriage, infertility

## Abstract

Background: This case–control study investigates whether miR-27a rs895819 A>G polymorphism is associated with an increased risk of recurrent pregnancy loss (RPL) in Caucasian Greek women. Methods: This study included 93 women with at least two unexplained miscarriages before the 24th week of gestation (RPL group) and 107 women with no pregnancy loss history (control group). The miR-27a rs895819 A>G polymorphism was detected using PCR amplification, followed by DraIII-HF restriction enzyme digestion. Results: The GG genotype was linked to a significantly higher risk of RPL (*p*-value = 0.00005), whereas the AA genotype was associated with a significantly lower risk (*p*-value = 0.00036). The AG genotype appeared more frequently in women with RPL (49.5% vs. 44.9% in controls), but the difference was not statistically significant (*p*-value = 0.5139). Conclusions: To our knowledge, this is the first study demonstrating that the miR-27a A>G polymorphism was significantly associated with a higher risk of recurrent miscarriage in Caucasian women. These findings provide evidence that the GG genotype may serve as a potential genetic marker for identifying women at higher risk of recurrent miscarriage, offering valuable insights for genetic counseling and reproductive medicine.

## 1. Introduction

Infertility is a major global health issue with profound effects on individuals and society. According to the latest definition by the American Society for Reproductive Medicine (ASRM), infertility is a disease, condition, or status of the reproductive system characterized by the failure to achieve a pregnancy after regular and unprotected sexual intercourse or the need for medical intervention [1]. Approximately 12% of reproductive-age couples worldwide experience infertility [2]. Over recent decades, fertility rates have declined significantly, with further decreases expected in the future [3]. In 85% of infertility cases, both male and female factors contribute, while the remaining 15% remain unexplained, a condition referred to as unexplained infertility [4]. Female infertility can result from advanced maternal age and medical conditions such as premature ovarian failure, polycystic ovary syndrome (PCOS), endometriosis, endometrial polyps, uterine fibroids, tubal obstruction, increased androgen levels due to adrenal hyperplasia or tumor and abnormalities in the uterine cavity which are also linked tο spontaneous miscarriages and preterm births [5]. Other factors that could affect female fertility include genital infections, obesity, smoking and exposure to various environmental pollutants [2,6]. Male factor infertility is influenced by multiple factors, with DNA fragmentation playing a crucial role in male reproductive capacity, leading to sperm DNA defects and ultimately increased aneuploidy rates [7,8,9,10,11]. Additional factors include genetic factors, oxidative and reductive stress, environmental toxins, and medications that impact the hypothalamic-pituitary-gonadal axis [12,13].

Recurrent pregnancy loss (RPL), defined by the ASRM and the European Society of Human Reproduction and Embryology (ESHRE) as two or more consecutive pregnancy losses before the 24th week of gestation, affects 1–2% of reproductive-age couples [14,15]. RPL is a complex and emotionally distressing condition, with no identified causal factor in 50% of cases [16]. The most common contributors include congenital or acquired uterine abnormalities, genetic factors (e.g., aneuploid embryos), endometriosis, autoimmune disorders (e.g., antiphospholipid syndrome and thyroid dysfunction, endocrine disorders (e.g., PCOS and vitamin D deficiency), thrombophilia factors, and reproductive tract infections [17,18,19,20,21,22,23,24]. While male factors have historically been overlooked in RPL research, recent studies highlight the role of high sperm DFI and epigenetic alterations in sperm DNA, both of which may contribute to recurrent miscarriage [8]. Increasing attention has also been given to the role of microRNAs (miRNAs) in idiopathic RPL [25].

MicroRNAs (miRNAs) are small, non-coding, single-stranded RNA molecules (22–24 nucleotides long) that regulate gene expression at the post-transcriptional level [26]. They influence key cellular processes such as cell division, differentiation, and apoptosis in both normal physiology and disease [27]. In the female reproductive system, miRNAs regulate ovarian function, steroid hormone synthesis, folliculogenesis, and oocyte maturation [28,29]. In pregnancy, trophoblast cells in the placenta produce miRNAs that modulate cell cycle regulation, differentiation, and apoptosis [30]. Recent studies have suggested that miRNA polymorphisms may be linked to RPL with functional single nucleotide polymorphisms (SNPs) in miRNAs potentially affecting their expression and maturation, increasing susceptibility to pregnancy complications [31,32].

The miR-27 family consists of miR-27a and miR-27b, which differ by a single nucleotide at the 3′ end [33]. miR-27a, located on chromosome 19, plays a role in gene regulation through its pre-miRNA hairpin structure [34]. The miR-27a rs895819 A>G polymorphism occurs in the stem-loop structure, which may alter miRNA processing by Dicer, affecting its maturation and function [35]. Studies indicate that G allele carriers have higher miR-27a expression levels than individuals with the AA genotype, which may have functional consequences [36]. Furthermore, miR-27a rs895819 has been associated with increased risk for colorectal cancer, breast cancer, gestational diabetes mellitus, and higher risk of recurrent abortions in different ethnic populations [37,38,39,40,41].

This study aims to investigate the possible correlation of miR-27a rs895819 A>G polymorphism in Caucasian women with recurrent pregnancy losses compared with a matched control group with at least one live birth and without any previous pregnancy losses. Ultimately, this study aims to assess the potential impact of the polymorphism as a novel genetic risk factor for recurrent pregnancy loss in Caucasian women.

## 2. Materials and Methods

### 2.1. Study Design

This prospective case–control study included 200 women. The recurrent miscarriage group (RPL group) included 93 women who attended the Recurrent Miscarriage Outpatient Clinic at the Alexandra Hospital, First Department of Obstetrics and Gynecology, Medical School of the National and Kapodistrian University of Athens. The control group included 107 women. The sample was collected in a three-year period until June 2024.

The inclusion criteria for the RPL group included women with at least two or more consecutive pregnancy losses before the 24th week of gestation. Women included in the control group should have at least one live birth without any prior pregnancy loss. The exclusion criteria included any endometrial or endocrinological pathology and/or any medical history of endometriosis, hydrosalpinx, thrombophilia disorders, autoimmune disorders, or chromosomal abnormalities. More specifically, chromosomal abnormalities were excluded via parental karyotyping, uterine and adnexal pathologies were excluded via conventional ultrasound scan, and endocrinological, immunological, and thrombophilia disorders were excluded via the routine recurrent pregnancy loss screening as suggested by the ESHRE 2022 guidance [14]. Furthermore, all women with abnormal semen analysis of the male partner were also excluded from the study. Patient characteristics, such as age and body mass index (BMI), were registered for the recurrent miscarriage group. The age and the BMI of the control group were matched to those in the recurrent miscarriage group.

The sample size calculation was based on the mean incidence of homozygosity and heterozygosity (GG + AG) for the polymorphism in the study and control groups of the existing published literature. The mean observed incidence was 64% for the recurrent pregnancy loss group and 44% for the control group. A power analysis was conducted, and results were estimated for an alpha value of 0.05 and a power of 80% to avoid type I and type II errors. With those values, the sample was calculated at 193 participants, divided into two groups, namely the RPL group (*n* = 92) and the control group (*n* = 101), with an enrollment ratio of 1.1. Since the existing literature referred to different racial populations and no study was conducted on Caucasian women, a retrospective post hoc power analysis was conducted for our specific sample, which validated a statistical power of 95% for the sample.

### 2.2. Ethical Approval

This study was conducted in accordance with the Declaration of Helsinki and approved by the Institutional Review Board of the Medical School of the National and Kapodistrian University of Athens with protocol identifier 56353 on the 26th of November 2020. All patients included in the study gave their informed consent for participation in the study. Furthermore, all patients included in the study gave their informed consent to publish the results.

### 2.3. DNA Extraction and rs895819 Polymorphism Genotyping

Peripheral blood samples were collected and processed for DNA extraction using the commercially available kit PureLink Genomic DNA Mini Kit (Invitrogen™, Life Technologies, San Francisco, CA, USA). The rs895819 polymorphism was detected using the Polymerase Chain Reaction (PCR) method.

The miR-27a rs895819 A>G polymorphism was identified using the following primers:

Forward: 5′ GAACTTAGCCACTGTGAACACCACTTG 3′;

Reverse: 5′ TTGCTTCCTGTCACAAATCACATTG 3′.

The amplified gene region was 182 bp in size, located between bases 13,835,450 and 13,836,631 on chromosome 19. The 27aF primer (27 bp long) amplifies the region between bases 13,836,450–13,836,476, while the 27aR primer (25 bp long) amplifies the region between bases 13,836,607–13,836,631. The 27aF primer contains a mismatch sequence in which the C base has been replaced by G.

PCR products were visualized using agarose gel electrophoresis. The PCR-amplified sequences were then incubated at 37 °C for 16 h with the DraIII-HF^®^ restriction enzyme (New England BioLabs, Ipswich, MA, USA) for polymorphism detection. The DraIII-HF^®^ enzyme cleaves the sequence at the following sites:

5′…CACNNN↓GTG…3′;

3′…GTG↑NNNCAC…5′.

Following incubations, electrophoresis of the digestion products was performed on a 3% *w*/*v* agarose gel with the DraIII-HF^®^ restriction enzyme. Based on the restriction enzyme cleavage pattern, three distinct banding patterns were observed, corresponding to different genotypes. The presence of a single fragment of 182 bp corresponds to the CC (GG) genotype, which represents homozygosity for the polymorphism. The presence of two fragments of 155 bp and 27 bp corresponds to the TT (AA) genotype, representing homozygosity for the wild-type allele. Finally, the presence of three fragments of 182 bp, 155 bp, and 27 bp corresponds to the TC (AG) genotype, indicating heterozygosity for the polymorphism.

## 3. Results

### 3.1. Baseline Characteristics

A total of 93 women with recurrent miscarriages and 107 controls were included in our study. The mean age was 33.98 for the RPL group and 34.02 for controls, while the mean values of body mass index (BMI) for both groups were 23.11 and 23.29, respectively. Both age and BMI did not differ between the two groups. Table 1 summarizes the baseline characteristics for both the recurrent miscarriage group and the control group.

### 3.2. Detection of rs895819 Polymorphism

**Figure 1:**
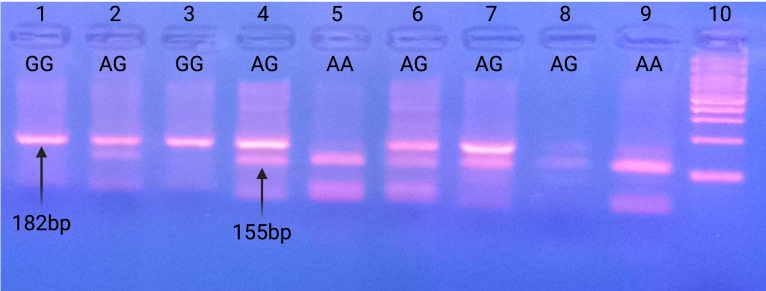
Detection of rs895819 polymorphism by gel electrophoresis. Lanes 1 and 3: Homozygosity for GG 182 bp. Lanes 2, 4, 6, 7, and 8: Heterozygosity 182 bp + 155 bp + 27 bp. Lanes 5 and 9: Homozygosity for AA 155 bp + 27 bp and Lane 10: 100 bp ladder.

The miR-27a rs895819 A>G single-nucleotide polymorphism (SNP) involves an A-to-G substitution. The PCR products generated were 182 bp in length. Following incubation with the DraIII restriction enzyme, digestion produced different fragment patterns based on the genotype. This enzyme recognizes a single cleavage site in the wild-type genotype (AA), resulting in DNA digestion that produces two fragments of 155 bp and 27 bp. The A to G substitution eliminates the recognition site, creating a different fragment pattern, specifically 182 bp, 155 bp, and 27 bp in heterozygous individuals (AG) and a single 182 bp fragment in homozygous individuals (GG). Figure 1 illustrates the detection of rs895819 polymorphism by gel electrophoresis.

### 3.3. Correlation of rs895819 Polymorphism and Recurrent Pregnancy Loss

In the RPL group, 24 women (25.8%) were found to be homozygous for the wild-type allele (AA), 46 women (49.5%) were heterozygous for the polymorphism (AG), and 23 women (24.7%) were homozygous for the polymorphism (GG). In the control group, 54 women (50.5%) were homozygous for the wild-type allele (AA), 48 women (44.9%) were heterozygous for the polymorphism (AG), and 5 women (4.8%) were homozygous for the polymorphism (GG).

The genotype frequency distribution suggests that the AG genotype occurs at a similar rate in both groups. However, a statistically significant difference was observed in the AA and GG genotypes. The AA genotype was significantly more frequent in the control group compared to women with RPL. In contrast, the GG genotype was significantly more frequent in women with RPL compared to the control group. The increased frequency of the AA genotype in the control group and the GG genotype in the RPL group was statistically significant (*p* = 0.00036 and *p* = 0.00005, respectively). The AG genotype appeared at a slightly higher rate in women with RPL than in controls (49.5% vs. 44.9%). However, this difference was not statistically significant (*p*-value = 0.5139).

Statistical analysis revealed that women homozygous for the miR-27a A>G polymorphism (GG) had a 6.703-fold higher risk of experiencing recurrent spontaneous miscarriage compared to those with the wild-type genotype (AA) (OR = 6.703, *p* = 0.0005, 95% CI = 2.432–18.47). Τhis association was statistically significant. Women who were heterozygous (AG) had a 1.203-fold increased risk of recurrent pregnancy loss compared to the wild-type genotype. However, this correlation was not statistically significant (OR = 1.203, *p* = 0.5139, 95% CI = 0.689–2.100). Finally, the AA genotype was 0.3 times less frequent in the RPL group compared to the control group, and this difference was statistically significant (OR = 0.341, *p* = 0.00036, 95% CI = 0.187–0.622). Table 2 demonstrates the genotype and allele frequencies of miR-27a rs895819 in the recurrent pregnancy loss group and control group.

## 4. Discussion

This study demonstrates that homozygosity for the miR-27a rs895819 polymorphism (GG) has a significantly higher risk for recurrent miscarriage compared to those homozygous for the wild-type allele (AA). Specifically, women with the GG genotype exhibited a 6.7-fold increased risk of having recurrent miscarriages (*p* = 0.00005), whereas women with the AA genotype had a significantly lower risk. Conversely, the AG genotype was not associated with recurrent miscarriage, as its frequency was similar in both the RPL and control groups. These findings suggest that the miR-27a rs895819 A>G polymorphism could potentially serve as a genetic risk factor for recurrent spontaneous abortions.

In recent years, increasing evidence has highlighted the role of genetic factors in recurrent pregnancy loss, with a particular focus on single nucleotide polymorphisms (SNPs) of microRNAs (miRNAs) [42]. MicroRNAs are small non-coding RNAs that regulate gene expression at the post-transcriptional level by targeting the 3’ UTR of mRNAs, thereby suppressing protein synthesis [43]. Scientific evidence suggests that dysregulated miRNA expression is involved in the pathophysiology of RPL. More analytically, microRNAs secreted by preimplantation embryos and blastocysts significantly influence endometrial receptivity during the apposition and adhesion phases by promoting cell migration and trophoblast cell attachment or by inhibiting adhesion [44]. Other key contributions of mRNAs are endometrial decidualization, placental development, and angiogenesis. Dysregulated mRNA expression has been linked to impaired placenta formation and increased risk for spontaneous abortions [45]. SNPs in miRNA genes may alter their function by modifying mRNA binding affinity, transcription, and maturation, potentially increasing the risk of RPL [46].

Genetic variations in the miR-27a gene have been linked to increased susceptibility to recurrent pregnancy loss due to implantation failure [47]. The rs895819 SNP leads to an A-to-G substitution in the miR-27a gene sequence. Several studies have investigated the association between this SNP and RPL risk in different populations. Our findings are consistent with previous studies, suggesting that the miR-27a rs895819 A>G polymorphism increases the risk of recurrent pregnancy loss.

For instance, Shaker et al., reported that both GA and GG genotypes were strongly associated with recurrent miscarriage in Egyptian women, even after adjusting the *p*-value [48]. Additionally, they demonstrated that the G allele was significantly overrepresented in the affected women, suggesting it as a risk allele for RPL. Similarly, a study conducted in China by Wang et al. found that the GG genotype and G allele frequencies were significantly higher in women with RPL compared to controls [49].

On the other hand, conflicting results have also been reported. Rah et al. found that both homozygosity (GG) and heterozygosity (AG) for miR-27a rs895819 polymorphism were associated with lower miR-27a expression levels, which appeared to protect Korean women from RPL. The researchers linked this protective effect of the G allele to higher circulating folate levels, as folate plays a critical role in pregnancy maintenance and reduces RPL risk [50]. Nevertheless, further research is required to confirm whether miR-27a influences recurrent pregnancy loss through folate metabolism. Additionally, a 2022 study by Kim et al., which included women from Korea, suggested that women heterozygous for the miR-27a rs895819 polymorphism (AG) were more likely to achieve a successful pregnancy, whereas AA genotype carriers had a higher risk of recurrent miscarriages. However, this genotypic difference was not statistically significant [51].

The present study adds to the paucity of published literature regarding the role of miR-27a rs895819 A>G polymorphism in recurrent miscarriages. To our knowledge, this is the first study demonstrating a significant association between miR-27a rs895819 A>G polymorphism and recurrent miscarriage in Caucasian women. The latter, in combination with the sufficient sample, constitute the strengths of this study. As far as limitations are concerned, it should be mentioned that our sample included only the Hellenic population, and further studies are needed to extrapolate the results of this study to the general population. Further studies are also needed to elucidate the exact pathophysiologic mechanism by which homozygosity for either the normal or polymorphic allele influences pregnancy outcome.

Further research is required to completely understand the underlying mechanism of recurrent miscarriages, even if this study has provided valuable insights into their genetic basis. Investigating the mechanisms of recurrent miscarriage, particularly the role of miR-27a in the pathophysiology of pregnancy loss, would enhance our understanding of the biological processes involved. Placental development, trophoblast invasion, and immunological regulation at the maternal–fetal interface are among the important pregnancy pathways that miR-27a is known to control [52,53]. Altered expression of miR-27a, due to the rs895819 SNP, could disrupt these processes, potentially leading to failure in implantation, impaired placental development, or abnormal immune responses, all of which could contribute to recurrent miscarriage. To better contextualize the potential biological pathways influenced by miR-27a, we incorporated predictive target analysis using publicly available bioinformatics tools such as TargetScan and miRDB. These analyses identified several putative targets of miR-27a that are functionally involved in key reproductive processes, including implantation (e.g., *ITGA5* and *VEGFA*), decidualization (e.g., *HOXA10*), and placental development (e.g., *FLT1* and *ENG*) [54]. Dysregulation of these targets has been previously associated with impaired endometrial receptivity, abnormal trophoblast invasion, and defective angiogenesis, contributing to pregnancy loss. Although experimental validation is still required, these predictive findings support the hypothesis that altered miR-27a function due to the rs895819 polymorphism may disrupt essential gene regulatory networks involved in early pregnancy maintenance [55].

Future research could use qRT-PCR to measure miR-27a expression levels in individuals with different genotypes (AA, AG, GG) to confirm our findings. If this method shows a link between miR-27a expression and recurrent miscarriages, it will strengthen the connection with the miR-27a rs895819 A>G polymorphism. qRT-PCR could also help us better understand how miR-27a affects important pregnancy processes like trophoblast invasion, placental development, and immune modulation.

While transcriptomic approaches such as RNA sequencing (RNA-seq) would offer a comprehensive view of the downstream gene networks influenced by miR-27a dysregulation, such analysis is currently beyond the scope of our available resources. Nonetheless, we acknowledge its potential value and consider it a priority for future investigation. In the interim, combining qRT-PCR with computational prediction tools (e.g., TargetScan and miRDB) may help identify and validate potential miR-27a target genes relevant to implantation and placental development.

By integrating molecular studies with clinical data, future research will be instrumental in clarifying how miR-27a variants contribute to RPL, potentially paving the way for genotype-guided risk assessment or targeted therapeutic strategies.

## 5. Conclusions

To our knowledge, this is the first study that demonstrates a significant association between miR-27a rs895819 A>G polymorphism and recurrent miscarriage in Caucasians. Based on our findings, combined with previous studies, we propose that the miR-27a rs895819 A>G polymorphism may be considered a potential genetic risk factor for RPL and can be used as a novel biomarker in the genetic counseling of infertile couples with recurrent abortions. However, it is important to mention that our results reflect the impact and incidence of the rs895819 A>G polymorphism in the Hellenic population, and further studies are needed to extrapolate our results to the general population.

## Figures and Tables

**Table 1:** Baseline characteristics in recurrent pregnancy loss patients and control group.

Variable	RPL Group (n = 93)	Control Group (n = 107)	*p*-Value
Age (Years)			
Mean (SD)	33.98 (5.881)	34.02 (5.849)	0.942
Median (Q1, Q3)	33 (30, 38)	33 (30, 38)	
BMI (kg/m^2^)			
Mean (SD)	23.11 (3.251)	23.29 (3.309)	0.681
Median (Q1, Q3)	22.60 (20.34, 24.99)	22.58 (20.42, 25.01)	

**Table 2:** Genotype and allele frequencies of miR-27a rs895819 polymorphism in recurrent pregnancy loss group and control group.

Genotype	RPL n (%)	Control n (%)	OR	95% CI for OR	*p*-Value
AA	24 (25.8%)	54 (50.5%)	0.341	[0.187–0.622]	0.00036 *
AG	46 (49.5%)	48 (44.9%)	1.203	[0.689–2.100]	0.5139
GG	23 (24.7%)	5 (4.7%)	6.703	[2.432–18.47]	0.00005 *
Total	93 (100%)	107 (100%)			

RPL: recurrent pregnancy loss, OR: odds ratio, CI: confidence interval. The asterisk (*) indicates significant *p*-values.

## Data Availability

The raw data supporting the conclusions of this article will be made available by the corresponding author upon request.
